# Unraveling the Roots of Selectivity of Peptide Affinity Reagents for Structurally Similar Ribosomal Inactivating Protein Derivatives

**DOI:** 10.3390/molecules21111504

**Published:** 2016-11-09

**Authors:** Deborah A. Sarkes, Margaret M. Hurley, Dimitra N. Stratis-Cullum

**Affiliations:** Biotechnology Branch, Sensors and Electron Devices Directorate, US Army Research Laboratory, Adelphi, MD 20783, USA; margaret.m.hurley12.civ@mail.mil (M.M.H.); dimitra.n.stratis-cullum.civ@mail.mil (D.N.S.-C.)

**Keywords:** bacterial display, biopanning, peptide, abrin, RIPs, biosensing, toxin, XPairIt, molecular modeling, docking

## Abstract

Peptide capture agents have become increasingly useful tools for a variety of sensing applications due to their ease of discovery, stability, and robustness. Despite the ability to rapidly discover candidates through biopanning bacterial display libraries and easily mature them to Protein Catalyzed Capture (PCC) agents with even higher affinity and selectivity, an ongoing challenge and critical selection criteria is that the peptide candidates and final reagent be selective enough to replace antibodies, the gold-standard across immunoassay platforms. Here, we have discovered peptide affinity reagents against abrax, a derivative of abrin with reduced toxicity. Using on-cell Fluorescence Activated Cell Sorting (FACS) assays, we show that the peptides are highly selective for abrax over RiVax, a similar derivative of ricin originally designed as a vaccine, with significant structural homology to abrax. We rank the newly discovered peptides for strongest affinity and analyze three observed consensus sequences with varying affinity and specificity. The strongest (Tier 1) consensus was FWDTWF, which is highly aromatic and hydrophobic. To better understand the observed selectivity, we use the XPairIt peptide–protein docking protocol to analyze binding location predictions of the individual Tier 1 peptides and consensus on abrax and RiVax. The binding location profiles on the two proteins are quite distinct, which we determine is due to differences in pocket size, pocket environment (including hydrophobicity and electronegativity), and steric hindrance. This study provides a model system to show that peptide capture candidates can be quite selective for a structurally similar protein system, even without further maturation, and offers an in silico method of analysis for understanding binding and down-selecting candidates.

## 1. Introduction

Biopanning using bacterial display technology has become an increasingly useful tool for the discovery and analysis of peptide capture candidates and recognition elements for both biotic and abiotic materials [1,2,3,4,5,6,7,8,9,10,11,12,13,14,15,16,17]. These peptide capture candidates have the potential to be utilized both on and off-cell for a variety of applications, such as biosensors, fuel cells, and bioactuators [18]. Combined with semi-automated Magnetic Activated Cell Sorting (autoMACS) methods, this technology has been employed for the rapid discovery of robust recognition elements with affinity towards potential biological threats and food toxins, such as *Bacillus anthracis* and Staphococcal enterotoxin B (SEB) [7,9,11,14]. After the initial rapid biopanning procedure to enrich for peptide capture candidates against the target of interest, the peptide ligands can be synthesized off-cell for further testing and immediate use or successfully matured to more robust, higher affinity synthetic peptide capture reagents using Protein Catalyzed Capture Agent (PCC Agent) technology [19,20,21] since these peptides are an alternative precursor for PCC strategies which otherwise require structural and sequence information for the target of interest [22,23]. Additionally, discovery in a bacterial peptide display system allows for direct use of peptide recognition elements while displayed on the cell surface of *Escherichia coli* (*E. coli*) bacteria. Unlike phage display biopanning techniques [24,25,26], a competing technology for peptide reagent discovery, the genetic material encoding the displayed peptide is directly amplified and maintained by the bound cells in bacterial display, which are still growing and dividing, creating an opportunity for use as a living material.

One of the most significant challenges in the development of small molecule affinity reagents to proteins, including peptide-based materials, is specificity. We aim to address this fundamental concern before fully relying on the affinity and selectivity of peptide capture reagents for specific targets, essentially replacing antibodies in a variety of applications that require more stable and robust reagents. Therefore, the focus of this work is to show that individual peptides from biopanning bacterial display libraries can be quite specific, even differentiating between structurally similar proteins. The Type 2 ribosome-inactivating proteins (RIPs) abrin and ricin were chosen for this study as a model system, utilizing their non-toxic derivatives, abrax and RiVax, respectively, due to their substantial structural homology and the extreme toxicity of abrin and ricin [27,28]. These RIPs contain two subunits: an A-chain that catalyzes the deadenylation of 28S rRNA to inhibit protein synthesis, and a B-chain that facilitates the uptake of the toxin into the cell [29,30]. The A-chains of ricin and abrin share 40% sequence identity, including the enzymatic active site, while the B-chains share 60% sequence homology [31,32]. The structures of these proteins are also remarkably similar, which can be visualized by overlaying their crystal structures [33]. The A-chains alone were used in the protein engineering strategies for creating RiVax, a ricin vaccine, and abrax, which was designed using the same approach to create a non-toxic derivative of abrin for study [27,34,35]. In addition to excluding the B-chain and therefore preventing uptake of the toxin into the cell, abrax and RiVax each contain two point mutations, Y80A and V76M on RiVax and homologous sites on abrax, to eliminate ribotoxicity and vascular leak-inducing ability, respectively [27,28]. Attempts to weaponize both abrin and ricin in the United States have been reported as recently as 2013 [36]. Using these similar toxin derivatives as a model system allows for a focused study in recognition of similar proteins that could also yield useful capture reagents for a weaponizable toxin.

Several antibodies against abrin currently exist, including a neutralizing antibody, D6F10, that rescues cells and protects mice from lethal doses of abrin [37,38,39,40], a potential immunotherapeutic antibody A7C4 that prevented toxicity and lethality in mice [41], and human antibodies isolated from phage display libraries [42]; however, peptide capture reagents are more thermally and chemically stable and could therefore be extremely useful for certain applications, such as toxin detection in extreme environments. Other alternatives to standard antibodies do currently exist for abrin or its derivatives. For example, Goldman et al. produced more thermally stable single domain antibodies (sdAbs) from llamas immunized with a commercial abrin toxoid. The high affinity binders in this work were specific for *Abrus* agglutinin, a protein related to abrin with much lower toxicity, but did not bind well to commercial abrin [27]. Aptamers also tend to be more stable alternatives to antibodies for use in biosensors. An abrin aptamer has also been discovered which does not cross-react with ricin in complex serum samples, but no consensus sequence was observed among the candidates, and understanding of how this selection is achieved is limited [43,44].

Despite long-term interest in the development of antibodies and other agents against these proteins, only a handful of studies probing the mechanism of neutralization exist for ricin or abrin, and details of the neutralized complex, including binding mode and location, are largely unknown. The existing experimentally determined epitopes have been of limited utility, as they have encompassed a somewhat broad swathe of the protein structure [37], or have consisted of scattered patches over the protein surface [45], or have varied widely between species [46]. Computational studies have largely focused on ricin and have included studies performing molecular dynamics simulations and simulated annealing as well as docking, dynamics and free energy determinants of a 29-mer oligonucleotide against the A-chain of ricin [47,48], as well as docking and pharmacophore model development of drug analogues from the Pubchem and Zinc databases against the ricin A-chain [49]. Recent work by Luo et al. has used molecular docking and dynamics simulations to study the complex formed between the combined ricin A- and B-chains and variants of the anti-ricin chimeric monoclonal antibody C4C13, and employed this detailed understanding to propose antibody mutations to affect binding affinity [50]. Sharma et al. used a variety of web-based bioinformatics tools to study possible DNA/RNA sequences for binding against both ricin and abrin, but provided no experimental validation and no details of the binding mode [51].

As a proven computational method, XPairIt is useful for the prediction of peptide affinity reagent interactions with target proteins as it incorporates flexibility, which has been demonstrated to play a key role in these systems [52]. XPairIt combines the PyRosetta docking software [53] with the NAMD package [54] through a Python programming interface. Additionally, XPairIt may be used to predict peptide–protein interactions when no a priori information of ligand structure (beyond sequence) or binding location is available [55,56]. Here the XPairIt protocol is used in global docking mode to scan the entire protein surface to pinpoint putative binding locations. This is similar in spirit to current energy-based methods for prediction of simple ligand binding sites [57] with the benefit of improved representation of ligand functionality and complex flexibility. This differs from previous simulation studies of ricin complexation in that the peptides used in this study are not derived from known binders, their location and mode of binding are unknown, and no experimental data (e.g., NMR) are available to guide the computation. Calculation of binding affinities for protein–peptide interaction is recognized as a challenging problem for a multitude of factors including the widely varied nature of the interactions, as well as the computational expense of accounting for conformational entropy contributions that may play an important role [58]. Although researchers continue to make strides toward this goal through a variety of informatics and algorithmic improvements [59], meaningful general quantitative calculation of peptide binding affinities across multiple protein targets has not been achieved. Even computational prediction of protein–peptide binding locations is recognized as difficult for identifying sites in novel proteins [60]. For this reason, work is underway to refine binding analysis of consensus sequences to propose future variants for improved selectivity and affinity (much as was performed in reference [50]), while the current study focuses on computational probing of the protein surfaces as presented to the individual Tier 1 peptides and the Tier 1 peptide consensus. Through this, we hope to computationally identify differences in binding sites between the two proteins to compare and contrast the RiVax and abrax surfaces in terms of binding pocket size and environment, including hydrophobicity and electrostatic potential, to further understand the binding specificity of the individual peptides and consensus.

To the best of our knowledge, no detailed computational work has been combined with experimental studies to investigate in detail the forces leading to the specificity of a ligand for one structurally similar RIP (or derivative) over another. Here we address this gap using peptide sequences obtained from the eCPX 3.0 bacterial display library [12], as well as highly selective consensus sequences, and analyze their interactions with abrax and RiVax. Preliminary results for bulk screening of peptides, after four rounds of biopanning to isolate abrax binding peptides, demonstrated significant enrichment of peptides with preference for abrax over RiVax [15]. In the current study, we extend our analysis to individual isolates for sequence analysis, elucidation of consensus sequences, and determination of individual peptide binding affinity and specificity. Noted among the peptides studied were three consensus families with slightly different affinity and specificity profiles. We further analyze the highest affinity, Tier 1 peptides, and their determined consensus, using the XPairIt docking protocol to determine likely binding locations of these peptides on abrax and RiVax, enabling better understanding of what is driving the selectivity and the role of the consensus sequence in binding affinity and specificity. Although neutralizing abrin was not a goal of this work, we also address the potential of our peptide capture reagents to bind at or near the active site of abrin. Together, the affinity and selectivity analysis and determination of binding location demonstrate that peptide capture agents can be selective for structurally similar proteins.

## 2. Results and Discussion

### 2.1. Initial Sequence Analysis of Abrax Binding Peptide Candidates

**Figure 1:**
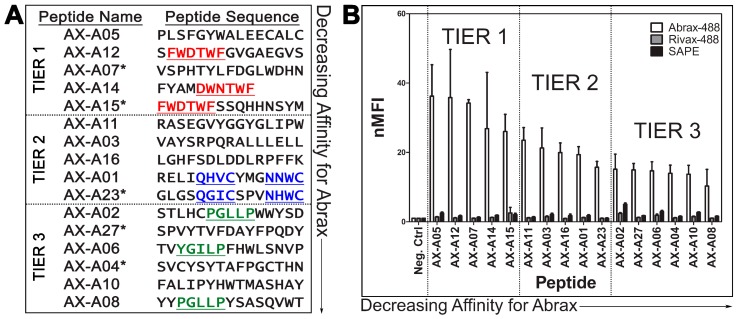
Three Consensus Families of Peptides Observed with Varying Binding Affinity: (**A**) Abrax binding peptide sequences with consensus families underlined and repeating sequences starred (*); and (**B**) FACS analysis of peptide binding to abrax-488 (white bars), RiVax-488 (gray bars), and Streptavidin-R-Phycoerythrin (SAPE, black bars). Plotted data are the average (bars) and standard deviation (error bars) of the normalized Median Fluorescence Intensity (nMFI) for three independent replicate experiments, normalized to the negative control cells. Actual values used for these calculations are shown in Tables S1–S3. The candidates are separated into three tiers based on abrax binding affinity and the focus of the remainder this work will include Tier 1 (highest affinity) peptides only.

As previously described [15], four rounds of biopanning the eCPX 3.0 bacterial display library for abrax binding peptides after an initial negative selection to remove RiVax binding peptides yielded new libraries of cells increasingly enriched for abrax binding peptides. Significant enrichment began at round 2 with 61.1% of the population bound to abrax and increased to 80.2% of the population by round 4. Individual isolates from the round 4 stock culture (100 colonies total) were DNA sequenced, translated, and the amino acid sequence analyzed for trends using Clustal W2, Clustal Omega, Kalign, and other techniques, including percent residue analysis, for amino acid comparison. At first glance, the isolated colony designated AX-A15 (Figure 1A) exhibits promising results since it was the most abundant peptide and accounted for 15% of the population analyzed. Four other sequences exhibited elevated frequency in the population characterized as well: AX-A07 (7%), AX-A27 (4%), AX-A04 (2%), and AX-A23 (2%). Overall, the repeating sequences accounted for 30% of the population sampled and analyzed.

Interestingly, two non-repeating sequences shared a consensus sequence with the most abundant peptide, AX-A15: FWDTWF, or slight variant, DWNTWF. These sequences were AX-A12 and AX-A14, respectively (Figure 1A, Tier 1). Two other consensus sequences were noted among non-repeating sequences as well: QXXCXXXNXWC and PGLLP, with slight variations among the peptides containing these motifs (Figure 1A, Tiers 2 and 3). These consensuses were noted in AX-A01 and AX-A23 for QXXCXXXNXWC and AX-A02, AX-A06, and AX-A08, for PGLLP (YGILP in the case of AX-A06). The analyzed subpopulation of 100 colonies did not display a uniform consensus but it was notable that the general population was enriched for aromatic and hydrophobic amino acid residues. This observation is consistent with the conclusions of London et al., that aromatic amino acids, as well as the hydrophobic amino acids leucine and isoleucine, are overrepresented in peptide hotspot residues [52]. Of the 100 colonies sequenced, 5% contained an HPQ motif, a known streptavidin binding motif, and were therefore predicted to bind streptavidin [61,62].

### 2.2. Characterization of Peptide Affinity and Specificity Using FACS and Further Sequene Analysis

From the sequence analysis in Section 2.1, all five candidates with repeating sequences were selected for further testing using Fluorescence Activated Cell Sorting (FACS). Additionally, the six candidates with non-repeating sequences that included the three consensus sequences described in Section 2.1 (Figure 1A, underlined) were analyzed along with an additional eight candidates chosen randomly in numerical order. Each candidate was compared and normalized to a negative control sample, which expressed the eCPX 3.0 display scaffold [12] and the P2X positive control peptide at the C-terminus, but not an experimental 15-mer peptide at the N-terminus, using normalized Median Fluorescence Intensity (nMFI) [14,63] (Figure 1B, Tables S1–S3). All candidates analyzed by FACS are listed in the sequence and affinity analysis results in Figure 1 and Tables S1–S3 except that three of the randomly chosen candidates that were analyzed using FACS are not shown because they demonstrated no binding to abrax or RiVax, with nMFI for abrax equal to or less than 1, but rather strong binding to streptavidin due to an HPQ motif [61,62]. Two additional sequenced candidates that were not tested for binding affinity using FACS but contain an HPQ motif are also expected to bind streptavidin and were therefore excluded from this analysis. The library was depleted of most streptavidin binders with an initial negative sort to prevent interference from the very powerful biotin-streptavidin binding interaction utilized in the biopanning procedure. According to FACS analysis of round 4, streptavidin binding peptides remained in the isolated round 4 population at a rate of about 7.4%, so 5 in 100 isolated colonies containing the streptavidin binding HPQ motif is not surprising or problematic [15].

The sixteen analyzed candidates with demonstrated binding to abrax were arranged in decreasing order of affinity and separated into three tiers (Figure 1). Note that each tier of peptides contains one of the three consensus sequences described in Section 2.1, which could be useful for creating pairs of reagents with different binding profiles. It is important to note that all three tiers of peptide candidates bind abrax, with higher affinity for abrax than the negative control proteins, RiVax and streptavidin. Note also that the Tier 3 peptides are not only of lower affinity but are also generally less specific, with higher binding affinity observed for both negative control proteins in most candidates as compared to the other two tiers of candidates. The candidates containing PGLLP were among the least specific, especially considering streptavidin binding, and the streptavidin magnetic bead used for sorting may have contributed to binding during the biopanning process, despite negative selection stringency that was imparted during the discovery process. Tier 2 peptides have similar specificity to Tier 1 peptides, but for the remainder of this study, we will focus on understanding the interactions driving selection of abrax over the very similarly structured protein RiVax in the highest affinity, Tier 1 peptides only.

The most frequent sequence in the 100 colonies that were analyzed, AX-A15, is potentially the most cross-reactive with RiVax, although Tier 3 peptide AX-A02 is a close second and has a lower standard deviation. Nevertheless, AX-A15 and AX-A12 contain the same consensus sequence, FWDTWF, with slightly different selectivity profiles, so it is interesting to look beyond the consensus to understand the contributions of the additional residues. AX-A14 contains a very similar consensus, DWNTWF, with only four additional residues, and yet it still has high affinity and selectivity for abrax, compared to the other tiers of peptides in this study. It could therefore be argued that the most critical residues of the consensus are XWXTWF, even if both versions additionally contain a negatively charged aspartic acid residue, although in different positions. It is also notable that AX-A05, which does not contain the FWDTWF consensus or the variant of it, has the highest affinity for abrax in this study. It does share similarly spaced aromatic, hydrophobic amino acid residues to the FWDTWF consensus, instead containing SFGYWA in the aligned region (Figure S1), suggesting that phenylalanine and tryptophan at these positions may play the most significant role in binding abrax. Peptide AX-A07 also had two of these aromatic, hydrophobic residues although with slightly different positioning, FDGLW (Figure 1A, Figure S1). Additionally, Clustal Omega alignment of AX-A07 with the FWDTWF consensus alone shows that the peptide is most similar to the Tier 1 consensus at the C-terminus, LWDHN in AX-A07 versus FWDTWF in the consensus, which only includes the tryptophan, not the phenylalanine, of peptide AX-A07 (Figure S1). The extent of enrichment of aromatic, hydrophobic residues (phenylalanine, tryptophan, and tyrosine) varies within the top tier, with three total residues for AX-A05 and AX-A07, four for AX-A12 (all of which are contributed by the consensus), and five for AX-A14 and AX-A15. Both AX-A07 and AX-A15 additionally each contain two of the positively-charged aromatic residue histidine, for five and seven total aromatic residues, respectively. Therefore, these results suggest that although aromatic residues are driving or directing the binding to abrax, affinity actually decreases to some extent when the number of aromatic residues increases. The least specific Tier 1 peptide, AX-A15, has the highest number of aromatic residues, so specificity may also be affected with an overabundance of aromatic amino acids, or perhaps the double histidine in particular for the case of abrax vs. RiVax binding. It is difficult to determine without knowing the binding location, necessitating further analysis.

### 2.3. Modeling Binding Location of Tier 1 Peptides on Abrax and RiVax Using the XPairIt Protocol

**Figure 2:**
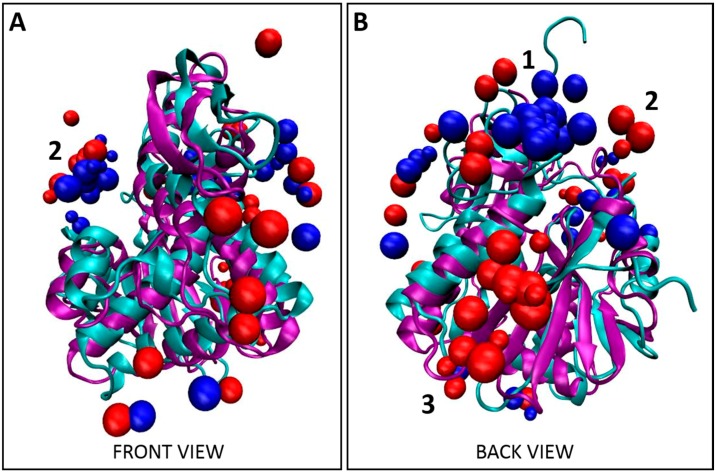
Localization of Tier 1 peptides. (**A**) Front view and (**B**) back view for the overlay of the top 10 decoys, as determined using XPairIt, of the abrax binding peptides A5, A12, A14, and A15 on RiVax (bound peptides: red spheres, protein: cyan ribbons) and abrax (bound peptides: blue spheres, protein: purple ribbons). Note the structural similarity between RiVax and abrax but disparity in predicted binding locations of the peptides. Binding locations 1–3 are numbered.

To better understand what is driving the selectivity of these Tier 1 peptides for abrax over the similarly structured and highly homologous toxin-derivative RiVax, a combined molecular docking/dynamics protocol was employed to study potential binding locations on each protein. This method corresponds to placing thousands of solvated peptide structures chosen from a molecular dynamics trajectory at thousands of different points on the (solvated, equilibrated) protein surface and subjecting each resultant structure to an iterative minimization/dynamics/repacking process, effectively testing peptide binding structure and energetics over the entire protein surface and allowing for binding-induced conformational changes. Using this ranking, the top 10 docked complex structures for each peptide were considered in our analysis. Typically, visual inspection and contact maps are sufficient to show the existence of a binding hotspot. Here we use a more formal analysis to look for binding location differences between abrax and RiVax, and for differences between peptides. Accordingly, the center of mass (COM) coordinates of the peptide backbone was accumulated for each decoy and the aggregation of all this coordinate data was used as input for the DBSCAN density-based clustering algorithm [64] as implemented in the scikit-learn toolkit [65]. Predicted top 10 binding conformations of the Tier 1 peptides on both abrax and RiVax are shown in Figure 2, Figure 4 and Figure S2. Discussion and analysis from here on will largely focus on peptides AX-A05, AX-A12, AX-A14, and AX-A15 (A5, A12, A14, and A15) as these sequences share significant overlap in sequence and binding motif (Figure S1). Candidate peptide AX-A07 (A07) did co-localize somewhat with the other Tier 1 peptides at one of the primary binding locations, (location 2), but demonstrated very limited sequence homology with the other Tier 1 peptides or the Tier 1 consensus (as described in Section 2.2 above), and more detailed analysis is therefore left to future work. It is clear both visually (Figure 2) as well as from clustering analysis (Table 1) that the binding locations of Tier 1 peptides, A5, A12, A14, and A15, are predicted to be quite different on abrax and RiVax, despite the structural similarity of these proteins. Possible binding locations for the Tier 1 peptides on abrax seem split between location 1 and location 2, which are rimmed by residues flagged in previous work as discussed below. For these peptides as a whole, 37.5% and 35% of the combined top 10 decoys bind to abrax at locations 1 and 2, respectively (Table 1). Peptide A5 had a stronger preference for location 1 on abrax than the three peptides (A12, A14, and A15) containing the Tier 1 consensus, with none of the top 10 decoys bound at location 2 (Table 1). Of the peptides containing the consensus, A14 had the strongest preference for location 1 while A12 and A15 had a preference for location 2 (Table 1). The bulk of the top 10 decoys for binding to RiVax bound to locations other than locations 1 and 2, with the most abundant binding site on RiVax designated as location 3 (Figure 2 and Table 1). The disparity in binding location of the top 10 decoys on abrax and RiVax shows great promise, especially since none of the top 10 decoys for these four peptides bind location 3 on abrax, the intended target.

**Figure 3:**
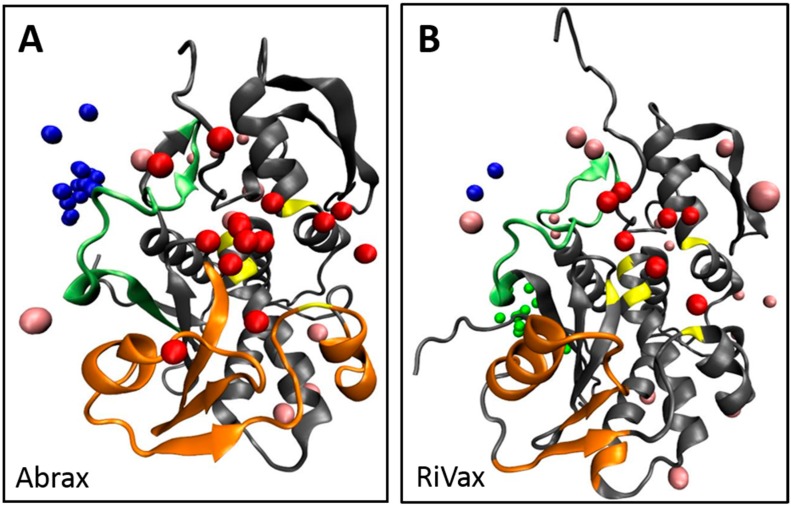
Structures of abrax (**A**) and RiVax (**B**), both shown in gray, with known epitope binding locations mapped in orange and the known solvent exposed loop, described by Compton et al., mapped in light green. Active site residues are shown in yellow. Top 10 decoys for Tier 1 peptide binders are colored by location to show location 1 (blue), location 2 (red), location 3 (green) and other binding locations (pink).

To put these three binding locations into context, we surveyed the literature. Location 1 is bordered by residues 34–43 which were flagged in ricin by Compton et al. as part of a solvent exposed loop, deletion of which leads to greater thermal stability and minimized protein aggregation [66]. Location 2 is rimmed at the top by the other side of this solvent exposed loop, and at the bottom by residues classified as a conserved neutralizing epitope by Lebeda et al. (residues 85 to 99) [67], and by residues (74–123) classified by Bagaria et al. as containing the core epitope for the neutralizing monoclonal antibody D6F10 in abrin [37]. From this, if our intention were to mature a peptide into a neutralizing agent for abrin, we might use peptides A12 or A15 as a precursor since they demonstrate a preference for location 2. To avoid the active site, A14 and A5, with stronger preference for location 1, would be more suitable for further maturation (Table 1). Both of these locations appear active in RiVax as well; however, the bulk of the binding appears to occur at a third location, with 37.5% of the top 10 decoys localized here on RiVax (location 3 in Figure 2, Table 1). Location 1 on RiVax is again bordered by the solvent exposed loop of Compton et al. [66], while location 2 in RiVax is an envelope of the solvent exposed loop, the active site of ricin (residues 80, 123, 177, 180, and 211), the epitope for the cluster 1 (residues 98–108) mAbs on ricin of Roy et al. [46], the epitope for camelid antibodies F5 and F8 of Rudolph et al. (residues 98–106, 113–117, and 154–156) [45], the epitope of the 6C2 (residues 102–106) mAb of Dai et al. [68] and the conserved neutralizing epitope of Lebeda et al. (residues 91–108) [67]. Location 3 does not correspond to a known epitope. Figure 3 shows the RiVax and abrax structures with peptide binders, with known epitope locations mapped. We note that the RiVax decoys show considerably more scatter across the surface of the protein, which may flag the weakness of the interaction. It has been noted on analysis of databases of high-resolution peptide–protein complex structures that the concept of “hot spot” residues contributing most of the binding energy applies to the peptide moiety as well as the protein [52,69]. Through XPairIt we have studied the per-residue contribution to the interface score for our Tier 1 peptides docked to abrax, and have found that this is indeed the case. For peptides A5, A14, A12, and A15 docked to abrax, the dominant contribution comes from a small subset of residues, mostly comprised of the aromatic residues (primarily W and F) in each sequence. We note that in A15 an additional contribution comes from the Histidine pair. This result is completely consistent with the nature of our consensus sequences, studied in the next section.

All computational work was performed, as stated, solely with A-chain protein structures to match experiment, with the goal of distinguishing between structurally similar proteins. To provide context for related structures containing both A- and B-chain proteins, we note that none of the primary binding sites (locations 1, 2, or 3) are situated in any portion of the A-chain surface expected to interact directly with a putative B-chain. This is visualized in Figure S3, utilizing crystal structures from PDB 1ABR [70] and PDB 2AAI [71]. Although we cannot rule out any possible interactions between the peptides and the B-chain itself in this analysis, short of performing additional simulation, we note that no primary binding location in either abrax or RiVax occurs in an area of the A-chain surface expected to interact directly with the main body of the B-chain. While a short section of the N-terminus of the B-chain of both abrin and ricin spans the ridge between location 1 and location 2, the residues involved are few and sufficiently hydrophobic to minimize potential steric hindrance with either binding site.

### 2.4. Modeling Binding Location of Tier 1 Consensus on Abrax and RiVax Using the XPairIt Protocol

**Figure 4:**
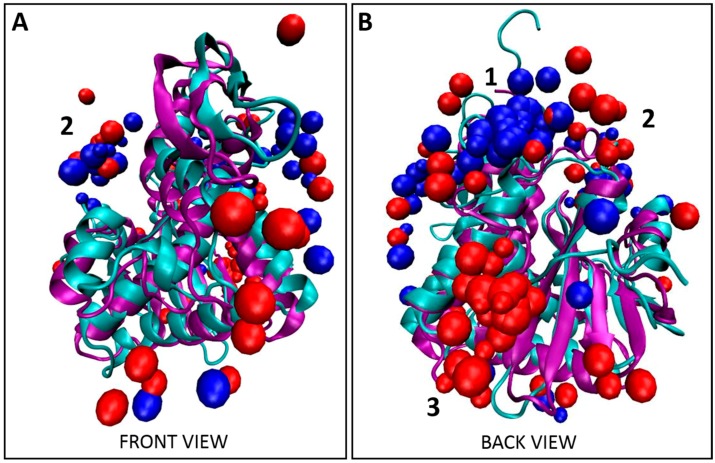
Co-localization of Tier 1 consensus with Tier 1 peptides. (**A**) Front view and (**B**) back view for the overlay of the top 10 decoys, as determined using XPairIt, of the abrax binding peptides A5, A12, A14, and A15 and the Tier 1 consensus on RiVax (bound peptides: red spheres, protein: cyan ribbons) and abrax (bound peptides: blue spheres, protein: purple ribbons). Note the structural similarity between RiVax and abrax but disparity in predicted binding locations of the peptides. Binding locations 1–3 are numbered.

Bioinformatic analysis of the five Tier 1 peptides in this study yielded the consensus FWDTWF (Figure S1). Two of these five peptides contained the consensus itself while one additional peptide contained a very similar variant, DWNTWF, and the remaining two peptides had at a minimum tryptophan and phenylalanine in similar positions, as described in Section 2.2 (Figure 1A). It was therefore important to determine if the Tier 1 consensus co-localized with the Tier 1 peptides, or if residues outside the consensus were also required for co-localization. Comparing Figure 2 and Figure S4 and Table 1 and Table 2, the predicted binding location of the FWDTWF consensus (and variant) did highly correlate with the binding locations of the individual Tier 1 peptides. Co-localization of the peptides is best demonstrated in Figure 4, which includes all decoys shown in Figure 2 and Figure S4. As with the individual peptides, and to a much greater extent, the bulk of the decoys for the Tier 1 consensus for binding to abrax, 70% (Table 2), shown in blue in Figure 4 and Figure S4, bind at location 1, with only 5% at location 2 and none at all at location 3. The remaining 25% of the decoys are scattered over the surface of the abrax. This binding location profile actually correlates better with the top binding peptide, A5, than with the individual peptides (A12 and A15) containing the Tier 1 consensus (compare Table 1 and Table 2). Peptide A14, with the DWNTWF variant, also correlates better with the consensus itself, potentially due to how few additional residues are present. The consensus has only 40% the length of peptide A5 (6 residues versus 15 residues, respectively) with a similar preference for location 1. The bulk of the decoys for binding to RiVax, 30% (Table 2), shown in red in Figure 4 and Figure S4, bind at location 3, with 25% at location 1 and 5% at location 2. Reducing the length of the binding peptide down to a consensus sequence has obviously had the effect of concentrating binding into location 1 on abrax, and, to a limited extent, on RiVax as well, although the primary binding location remained location 3. Fully 40% of the decoys for binding RiVax are dispersed over alternative locations. Clearly, for the consensus sequences, and for the Tier 1 peptides themselves as well, binding in abrax is more highly localized than in RiVax. The FWDTWF consensus seems to be enough to direct the binding location and provide selectivity for abrax over RiVax, so it would be an excellent candidate for further maturation into PCC agents to increase stability, affinity, and selectivity. It seems that the additional residues on peptides A12, A14, and A15 are contributing to the binding at the secondary site, location 2, since only 5% of the top 10 decoys for the Tier 1 consensus localize there while 35% of the collective Tier 1 peptides themselves are directed to location 2. This is especially the case for A12 and A15, for which 60% and 50% of the top 10 decoys were directed to location 2, respectively (Table 1). Using the consensus sequence, or variant, for further maturation could therefore yield a more specific peptide capture reagent.

### 2.5. Discussion of Electrostatics, Pocket Size, and Pocket Environment for Abrax and RiVax

**Figure 5:**
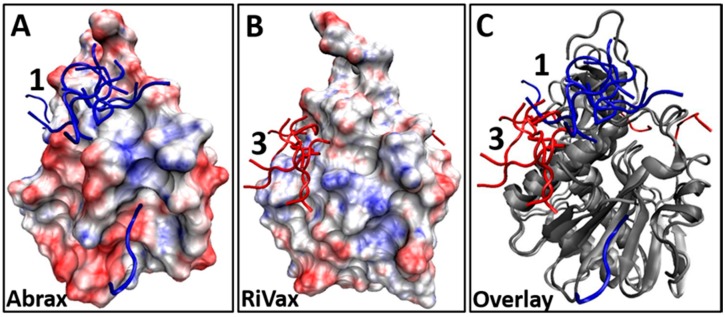
(**A**,**B**) Surface representation showing electrostatic potential (red = electronegative, blue = electropositive) and binding locations of the top 10 decoys for Tier 1 consensus sequence FWDTWF binding, found using XPairIt, to: (**A**) abrax (top 10 decoys are blue sticks); and (**B**) RiVax (top 10 decoys are red sticks); (**C**) Overlay of the abrax and RiVax images from (**A**) and (**B**) using ribbon protein backbone (in gray for both abrax and RiVax) to show that the binding locations of the consensus are different.

The disparity in binding location of the Tier 1 and consensus peptides on abrax and RiVax raises questions about what differences between these specific locations on the two proteins could be driving these interactions. It is known that electrostatics play an important role in the function of the ricin A-chain, but that this varies widely between ribosomal inactivating proteins [72]. We therefore examine the electrostatic potential surfaces for these two proteins, since structural similarity and 40% sequence identity leaves plenty of room for discrepancies in overall charge on the protein surface [27,28,32]. A surface representation of abrax and RiVax with electrostatic potential mapping is shown in Figure 5 (A and B, respectively). This is shown from the perspective of looking down into binding location 1 (the most popular binding location in abrax) with binding location 3 (the most popular binding location for RiVax) off to the left hand side. Backbone structures for the bound consensus sequence FWDTWF are shown to demonstrate context and separation. Clearly, despite high structural similarity and significant sequence homology, the electrostatic potential differs between these two proteins.

We look to further quantify differences in the protein binding locations using alternative measures. In studying the structural basis of protein–peptide interactions, London et al. concluded that peptide affinity reagents tend to bind to the largest pocket on the protein surface [52]. We characterized our binding locations for both abrax and RiVax using Fpocket software for protein cavity detection and characterization and determined that this is indeed true in our case. A visualization of the pockets at binding locations 1–3 is shown in Figure S5 while the pocket sizes are given in Table 3. In abrax, location 1 (the most occupied binding site according to our simulations) is clearly the largest of the three binding locations, and is in fact nearly 50% larger than location 3 (where no binding occurs). In RiVax, the opposite is true: location 3 is clearly the largest of the three pockets. It should additionally be noted that the pocket sizes are more uniform in RiVax than in abrax, and are indeed more evenly occupied according to our global check. These calculated pocket sizes are consistent with information gleaned from molecular dynamics simulations of the individual proteins. Dynamics simulations show, for example, hindrance of location 3 by the N-terminus of abrax, which is not seen in RiVax. Conversely, location 1 of RiVax is partially obstructed by an immobile alpha-helix which is somewhat elongated in comparison to the abrax structure.

There is however more to peptide–protein binding than the dictates of topology and it is conceivable from the large number of aromatic residues in the Tier 1 peptides and their consensus sequence that hydrophobic effects play an important role. We therefore use Fpocket to analyze an additional parameter to characterize the three locations, the mean local hydrophobicity density (Table 4). This descriptor provides a normalized measure of localization of hydrophobic character within a pocket, rather than a gross measure of overall hydrophobicity. Here is where we see an interplay between motive (hydrophobicity) and means (binding pocket volume) that can explain the trends shown in Table 1 and Table 2 and Figure 2, Figure 4 and Figure S4. Binding location 1 on abrax has a high hydrophobic density in addition to being the largest pocket, and is accordingly the most highly occupied binding location according to the global docking scan. Alternatively, binding location 3 on abrax has the lowest hydrophobic density in addition to being the smallest pocket of the three, and global docking shows no binding occurring there at all. Similarly, location 3 on RiVax is the largest of the three locations and has a reasonably high mean local hydrophobic density. Location 1 (which is also a well-represented binding site) has a high hydrophobic density but is somewhat smaller, which may explain, in part, why this location is more highly represented in the shorter consensus sequences than in the longer Tier 1 sequences (Table 1 vs. Table 2). Location 2 on RiVax has a low hydrophobic density and is poorly occupied, as expected.

**Figure 6:**
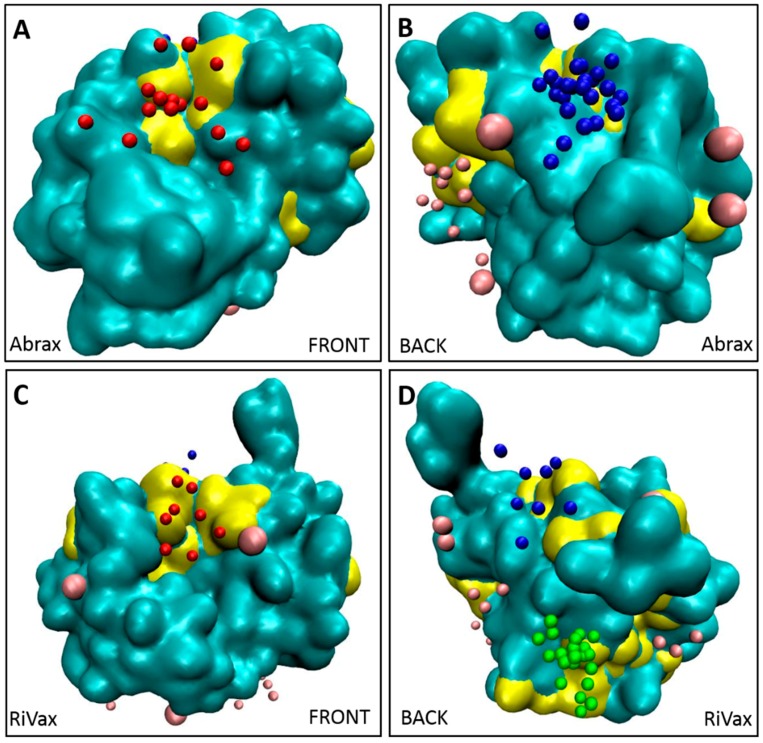
Clusters of hydrophobic residues from the Kyte-Doolittle scale (four or more in a row), colored in yellow, correspond to primary binding locations for both: abrax (**A**,**B**); and RiVax (**C**,**D**). Other residues of the protein are shown in cyan. Top 10 decoys for Tier 1 peptide binders (A5, A12, A14, and A15) are colored by location to show location 1 (blue), location 2 (red), location 3 (green) and other binding locations (pink). Front views of the proteins are shown in (**A**,**C**); and back views of proteins are shown in (**B**,**D**).

We further demonstrate this interplay between pocket size and hydrophobicity in Figure 6, where our binders are again shown in the context of the Kyte Doolittle hydrophobicity scale. Here any sequence of four or more hydrophobic residues in a row is marked in yellow on the protein surface. We see that this corresponds almost exactly to our flagged locations on both proteins and accounts in some measure for the high degree of localization in binding to abrax. Note especially the hydrophobic patch at location 3 on RiVax visualized in Figure 6D, but not on abrax in Figure 6B, which is consistent with the lack of binding at location 3 on abrax.

## 3. Materials and Methods

### 3.1. Expression and Purification of Abrax and RiVax

The abrax and RiVax recombinant proteins used in this work were expressed and purified from pET28a(+) plasmids obtained from Edgewood Chemical and Biological Center (ECBC, Aberdeen, MD, USA) using methods similar to their published protocol for abrax [27] and a similar published protocol for RiVax [35]. For growth, BL-21 (DE3) *E. coli* (Bio-Rad, Hercules, CA, USA) containing each plasmid were grown in Luria Bertani Miller Broth (for abrax) or Terrific Broth (for RiVax) containing 30 μg/mL kanamycin (LB Kan_30_ and TB Kan_30_, respectively). For induction of protein expression, 1 mM isopropyl-B-d-thiogalactopyranoside (IPTG, Sigma, St. Louis, MO, USA) was added and the cultures were incubated overnight (for abrax) or for 4 H (for RiVax) at room temperature (~22 °C) shaking at 225 RPM. Cell lysis was achieved using a microfluidizer with the cell pellet resuspended in Phosphate Buffered Saline (PBS, for abrax) or 20 mM sodium phosphate pH 8.0, 500 mM sodium chloride, 20 mM imidazole (for RiVax) and the proteins were purified from the supernatants using 5 mL HisTrap Crude FF columns (GE Healthcare Life Sciences, Pittsburgh, PA, USA), eluting with increasing imidazole to 500 mM, in PBS (for abrax) or 20 mM sodium phosphate pH 8.0, 500 mM sodium chloride (for RiVax), with high observed purity in the final sample. Abrax was buffer exchanged into PBS while RiVax was buffer exchanged into 20 mM sodium phosphate pH 8.0, 500 mM sodium chloride for further manipulation or 25 mM Citrate-Phosphate 1 M NaCl pH 6.0 for long-term storage at −80 °C. Purified abrax and RiVax proteins were biotinylated for use in biopanning using No-Weigh Sulfo-NHS-LC-Biotin (Thermo Scientific, Rockford, IL, USA) and labeled for use in FACS experiments using Dylight 488 NHS Ester (Thermo Scientific), following manufacturer’s instructions, in their working buffers: PBS for abrax and 20 mM sodium phosphate pH 8.0, 500 mM sodium chloride for RiVax.

### 3.2. Discovery and FACS Analysis of Abrax Binding Peptides

Similarly to previously described protocols [14,15], four consecutive rounds of biopanning were performed using the autoMACS^®^ Pro Separator (Miltenyi Biotec, San Diego, CA, USA) to enrich abrax binding peptides from a frozen eCPX 3.0 bacterial display library stock (CytomX Therapeutics, San Francisco, CA, USA [12]) containing approximately 1 × 10^11^ cells and pre-depleted of streptavidin binding peptides [7,8,9,14]. A negative sort for RiVax, round 0, was first performed as described in Sarkes et al. (2016) [15], to help deplete the library of peptides with affinity for this structurally similar protein. After initial confirmation by FACS that the enrichment of abrax binding peptides was successful in these four rounds of biopanning [15], 100 individual isolates from a spread plate (individual colonies grown overnight in a petri dish containing Luria-Bertani Miller Broth (LB, Fisher Scientific, Pittsburgh, PA, USA), agar (Fisher Scientific), and 25 μg/mL chloramphenicol (Sigma) were DNA sequenced (Genewiz, Frederick, MD, USA) to determine the amino acid sequence of the displayed peptide. Sequences were analyzed and aligned using Clustal Omega [73] and Jalview with Clustal_X Windows interface [74,75]. A subset of sequenced isolates was analyzed by FACS using the methods described in Sarkes et al. (2015) [14]. The subset for analysis was down-selected by testing all 5 repeating sequences (see Figure 1A, starred) and 3 non-repeating sequences that included consensus sequences noted in this set of repeat candidates, then extended to include 11 additional non-repeating colonies that either displayed other observed consensus sequences (see Figure 1A, underlined) or were chosen randomly in numerical order. Each isolate from this set was compared to a negative control sample, which expressed the eCPX 3.0 display scaffold and the P2X positive control peptide at the C-terminus, but not an experimental 15-mer peptide at the N-terminus, using normalized median fluorescence intensity (nMFI). The MFI of the total population for each sample was divided by the MFI of the negative control cell population incubated with the same fluorophore such that the nMFI of the negative control is always 1 and a binding event is therefore demonstrated by an nMFI greater than 1 [14,63]. The mean, standard deviation (SD), and standard error of the mean (SEM) of three independent experiments were calculated and the mean and SD were graphed using Prism Software (GraphPad, La Jolla, CA, USA).

### 3.3. Determination of Binding Location Using XPairIt Docking Protocol and Analysis Using Fpocket

The RiVax structure was taken from an existing X-ray diffraction structure (PDB 3SRP) [34] and minor repairs made: a missing short 5-residue sequence at the N-terminus was added using the Molefacture Plugin of VMD [76] before starting molecular dynamics. The abrax A-chain structure was obtained by making proper mutations to the X-ray diffraction structure of the abrin A-chain (PDB 1ABR) [70]. All structure setup was performed with VMD [76]. Structures were protonated, solvated with TIP3P water and a box padding of 15 Å on each edge of the molecular dimensions, and the resulting periodic box was charge-neutralized. The system was minimized and isothermal-isobaric ensemble (NPT) dynamics performed for 10 ns at 300 K and 1 atm using the CHARMM27 forcefield [77] to produce a starting structure for docking/dynamics simulations. A similar procedure was followed for all peptides, with the exception of starting structures which were built from sequence. Combined docking/dynamics simulations were performed using the XPairIt protocol with the Global Docking Method as outlined in reference [55]. This method takes thousands of solvated peptide structures chosen from a molecular dynamics trajectory, places them at thousands of different points on the (solvated, equilibrated) protein surface and subjects each resultant structure to an iterative process involving Rosetta docking and repacking, NAMD molecular dynamics and minimization and again Rosetta repacking. The resulting docked structures (or decoys) are ranked by total energy to remove high energy structures and then re-ranked by interface energy to look for favorable interactions within the complex. Center of mass (COM) coordinates of the peptide backbone were accumulated for each decoy using the center of mass function in VMD [76]. The aggregation of all this coordinate data was used as input for the DBSCAN density-based clustering algorithm [64] as implemented in the scikit-learn toolkit [65]. A minPts (minimum cluster size) value of 2 was used, and a variety of epsilon values were assessed for our cluster analysis. Results are presented for an epsilon value of 7.0. Careful assessment of PDB2PQR and APBS were used to calculate the electrostatic surface potential of each protein [78,79]. Additional analysis of the binding locations was performed using the Fpocket package of Guilloux et al. [80]. All visualization was performed with VMD [76]. The hydrophobicity scale of Kyte and Doolittle was used and quickly mapped with the ProtScale tool of the ExPASy Bioinformatics Resource Portal [81].

## 4. Conclusions

The primary objective of this work was to demonstrate that peptide capture agents are capable of selectivity of very similar targets. While previous work with biopanning of bacterial display libraries for peptide capture reagents demonstrated great promise, the approach had not been challenged to distinguish between targets characterized by high structural similarity. Non-toxic derivatives of the structurally similar, highly homologous plant protein toxins abrin and ricin, namely abrax and RiVax, respectively, were chosen as a model system [27,28]. Employing rapid, robust biopanning techniques with the eCPX 3.0 bacterial display library and autoMACS, numerous peptide capture candidates were discovered with impressive selectivity for abrax over the structurally similar protein RiVax. A subset of these candidates demonstrated three consensus families with slightly different affinity and specificity profiles. The top tier consensus sequence was FWDTWF, with a slight variant, DWNTWF, also noted in a single isolate, AX-A14. Focusing on the top binding “Tier 1” peptides, including those with the FWDTWF consensus, we used the XPairIt docking protocol to show clear differences in the likely binding locations of these peptides on abrax and RiVax, enabling better understanding of what is driving the selectivity. The Tier 1 consensus primarily contains aromatic, hydrophobic residues, and hydrophobicity clearly plays a driving role in determining binding locations on these two proteins. This trend is consistent with the finding of London et al. that aromatic amino acids, as well as the hydrophobic amino acids leucine and isoleucine, are overrepresented in peptide hotspot residues [52]. Stronger binding (experimentally) is seen in the abrax protein, where several large, locally hydrophobic pockets are available for peptide binding. Weaker binding (experimentally) is seen in RiVax, where scattered docking results coincide with a more general spread of hydrophobic residues over the protein surface. In addition to hydrophobicity, binding pocket volume and steric hindrance played a role in the selectivity of Tier 1 peptides, and their consensus sequence, for abrax over RiVax, especially at the abrax binding hotspot we have labeled location 1 in Figure 2, Figure 3, Figure 4, Figure 5 and Figure 6 and Figures S2–S5. Although location 1 was the primary binding location for abrax, it was a poor location for binding to RiVax, despite their structural similarity. Instead, location 3 was the primary binding location on RiVax (Figure 2, Figure 3, Figure 4, Figure 5 and Figure 6, Table 1 and Table 2, and Figure S4), although the peptides bound here with much lower affinity than the peptides bound at location 1 on abrax (Figure 1B, Tables S1 and S2). On abrax, the Tier 1 consensus sequence better localizes to a single binding location, location 1, than do the individual peptides that contain it. As compared to A12 and A15, more focused localization of peptide A14, with the DWNTWF consensus variant and only four additional residues, also highlights that removal of residues outside the consensus further concentrates binding to location 1. More localized binding demonstrates that the consensus itself could be an excellent precursor for maturation, and the overlay of our models with the full abrin and ricin proteins (Figure S3) demonstrates that the predicted binding sites would remain exposed with the addition of the B-chain, allowing the potential for maturated peptides to recognize the full toxin. Additional residues outside of the consensus may have the ability to improve specificity, but the additional residues found in peptides A12, A14, and A15 do not help lock in binding to location 1 according to our computational model. Peptide A5, with limited similarity to the Tier 1 consensus, is also an excellent candidate based on its high experimental affinity and selectivity, as well as its more targeted binding localization within abrax, according to our computational model. Together, these results demonstrate that peptide capture candidates can be rapidly and reliably screened on-cell to produce selective peptide affinity reagents. These peptides could be used on-cell for recognition, or synthetically produced off-cell for use in biosensors. Incorporating computational analysis strategies in the early stages of affinity reagent development, before off-cell production and analysis of peptides, is a promising approach to reduce time spent optimizing assays, such as Enzyme-Linked Immunosorbent Assay (ELISA) and Surface Plasmon Resonance (SPR), for screening, characterization, and down-selection of potential candidates for maturation. Our future goals include off-cell production of the Tier 1 consensus and variant, more focused docking studies of the consensus sequences (including Tier 2 and 3 consensus sequences) to flag more atomistic level interactions driving specificity, and further maturation into chemically and thermally stable PCC agents to produce truly robust peptide-based capture agents against abrax for testing against the abrin toxin itself.

## Figures and Tables

**Table 1:** Percent of Top Ten Decoys from XPairIt Analysis at Each Binding Location on Abrax or RiVax (Shown in Figure 2) for Tier 1 Peptides A5, A12, A14, and A15.

Protein Target (& Peptide Bound)	Binding Location 1	Binding Location 2	Binding Location 3	Other Binding Location
**Abrax:**				
All ^1^	37.5%	35%	0%	27.5%
AX-A15	20%	50%	0%	30%
AX-A12	10%	60%	0%	30%
AX-A14	50%	30%	0%	20%
AX-A05	70%	0%	0%	30%
**RiVax:**				
All ^1^	5%	17.5%	37.5%	40%
AX-A15	0%	0%	20%	80%
AX-A12	0%	30%	50%	20%
AX-A14	10%	10%	60%	20%
AX-A05	10%	30%	20%	40%

^1^ Includes top 10 decoys for A5, A12, A14, and A15 combined.

**Table 2:** Percent of Top Ten Decoys from XPairIt Analysis at Each Binding Location on Abrax or RiVax (Shown in Figure 4) for the Tier 1 Binding Consensus, FWDTWF.

Protein Target	Binding Location 1	Binding Location 2	Binding Location 3	Other Binding Location
Abrax	70%	5%	0%	25%
RiVax	25%	5%	30%	40%

**Table 3:** Pocket Volume (in cubic angstroms) at Each Binding Location on Abrax or RiVax (Shown in Figure S5) for Tier 1 Peptides A5, A12, A14, and A15 Determined Using Fpocket.

Protein Target	Binding Location 1	Binding Location 2	Binding Location 3
Abrax	**3162** ^1^	2281	2045
RiVax	2366	2648	**2951** ^1^

^1^ Bold font denotes larger pocket at location consistent with predominant binding location.

**Table 4:** Mean Local Hydrophobicity Density at Each Binding Location on Abrax or RiVax (Shown in Figure S5) Determined Using Fpocket.

Protein Target	Binding Location 1	Binding Location 2	Binding Location 3
Abrax	11.36	8.4	4.7
RiVax	10.13	4.17	7.38

## Supplementary Materials

Supplementary materials can be accessed at: http://www.mdpi.com/1420-3049/21/11/1504/s1.
