# Supplementary material for: Unraveling the Roots of Selectivity of Peptide Affinity Reagents for Structurally Similar Ribosomal Inactivating Protein Derivatives
Source: Molecules. 2016 Nov 9;21(11):1504. doi: 10.3390/molecules21111504 (PMC6272918; doi:10.3390/molecules21111504)
Supplement: Supplementary file 1 [file molecules-21-01504-s001.pdf]

Supplementary Materials: Unraveling the Roots of Selectivity of Peptide Affinity Reagents for Structurally Similar Ribosomal Inactivating Protein Derivatives

Deborah A. Sarkes, Margaret M. Hurley and Dimitra N. Stratis-Cullum

S.1. Supplementary Figures

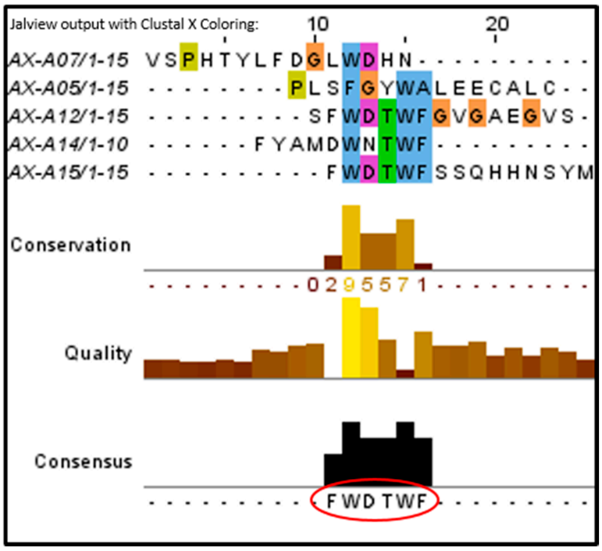

Figure S1. Clustal Omega alignment and Jalview analysis of Tier 1 peptides AX-A05, A07, A12, A14, and A15. Clustal X default coloring is used. The Tier 1 consensus, FWDTWF, is circled in red.

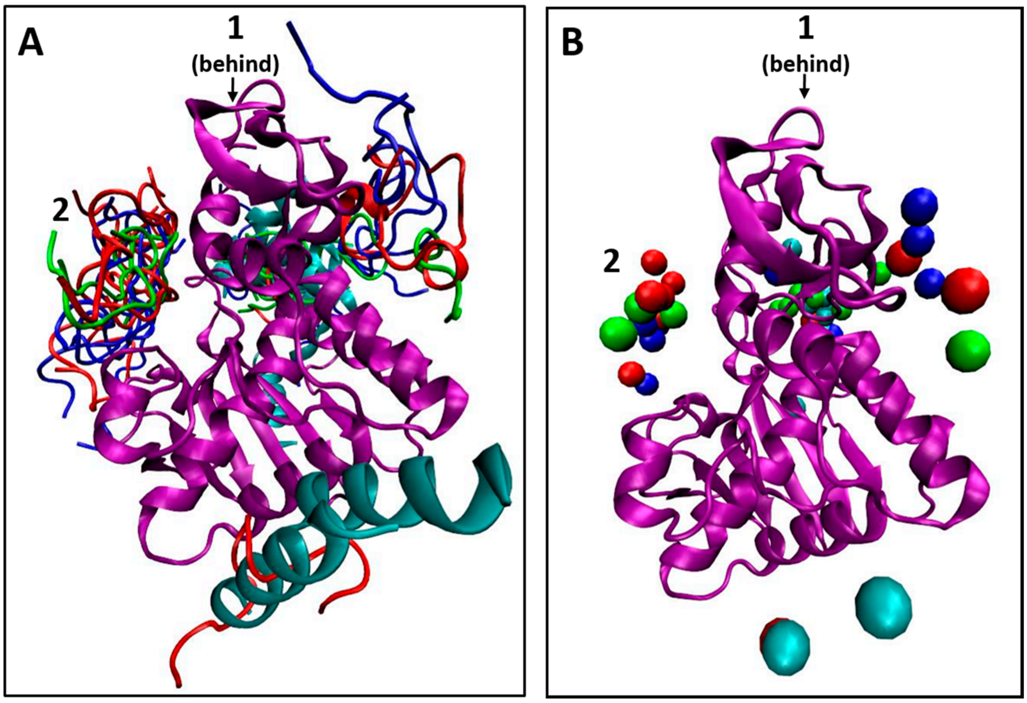

Figure S2. Overlay of the top 10 decoys for binding to abrax, as determined using XpairIt, of the abrax binding peptides A5 (blue), A12 (red), A14 (green), and A15 (cyan). The image in (**A**) shows peptide structure while (**B**) shows center of mass for each peptide. Binding locations 1 and 2 are numbered.

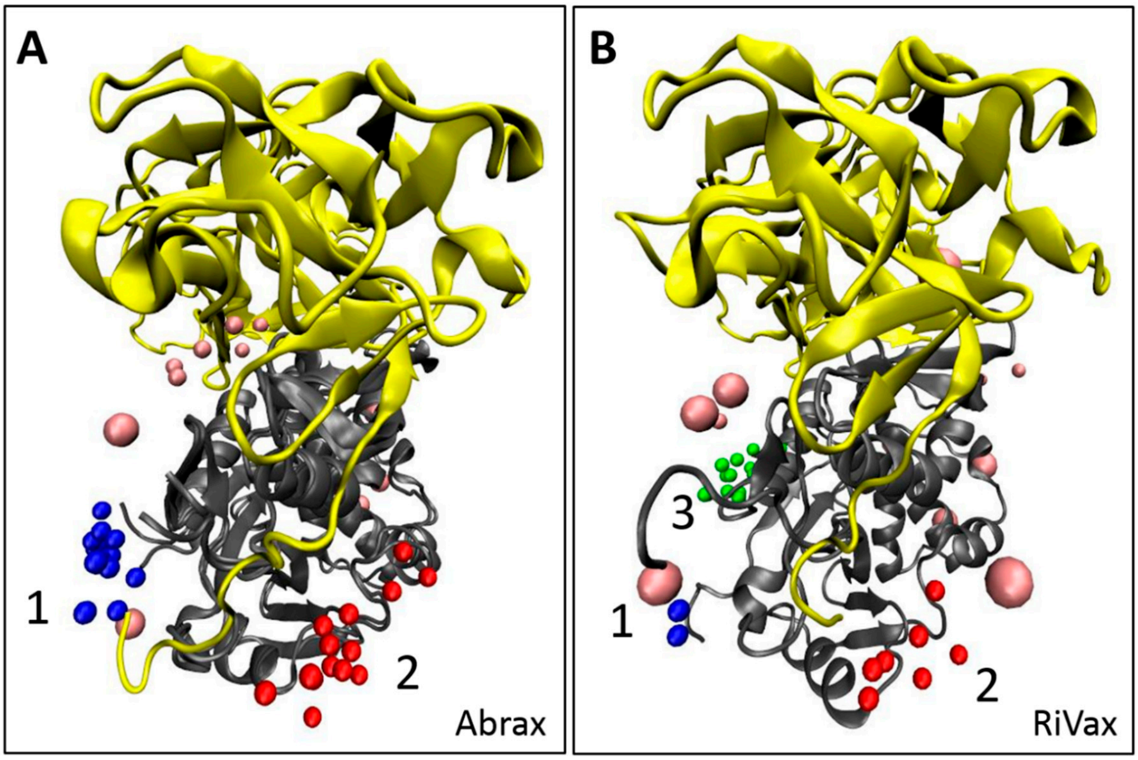

Figure S3. Overlay of abrin onto abrax (**A**) and ricin onto RiVax (**B**) docking results. The abrin and ricin structures are taken from PDB 1ABR [70] and PDB 2AAI [71]. The A chain of all proteins are visualized in gray. The B chain of abrin and ricin is visualized in yellow. Docking decoys are colored by cluster at location 1 (blue), location 2 (red), location 3 (green), and unclustered (pink).

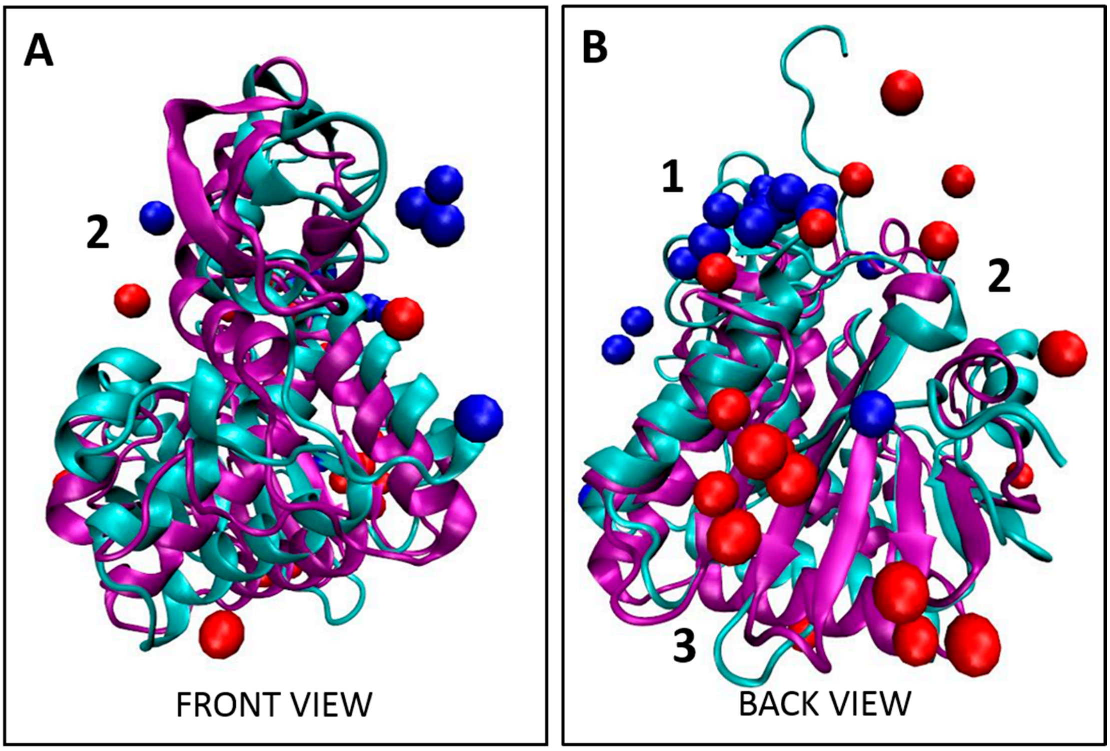

Figure S4. Localization of Tier 1 consensus. (**A**) Front view and (**B**) back view for the overlay of the top 10 decoys, as determined using XPairIt, of the Tier 1 abrax binding consensus on RiVax (bound peptides: red spheres, protein: cyan ribbons) and abrax (bound peptides: blue spheres, protein: purple ribbons). Note the structural similarity between RiVax and abrax but disparity in predicted binding locations of the peptides. Binding locations 1–3 are numbered.

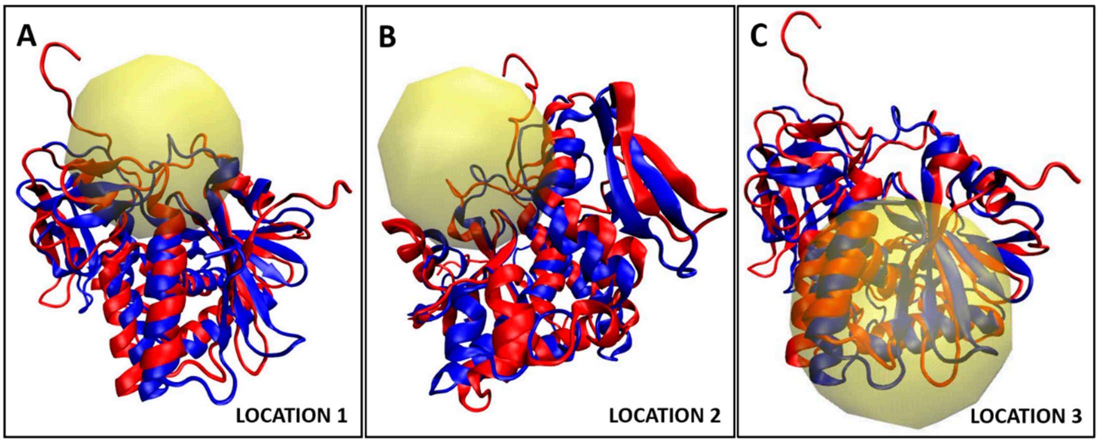

Figure S5. Visualization of pocket size and environment for binding locations 1–3 on abrax (blue) and RiVax (red) as determined using Fpocket software. Pocket volume shown in yellow at (**A**) location 1, (**B**) location 2, and (**C**) location 3. See Table 3 for quantitative analysis of pocket volume.

S.2. Supplementary Tables:

Table S1. FACS analysis of peptide binding to abrax-488. The normalized Median Fluorescence Intensity (nMFI) for three independent replicate experiments (R1–R3), normalized to the negative control cells, is shown along with the mean, standard deviation (SD), and standard error of the mean (SEM) for this data set. This data is plotted in Figure 1.

Peptide	Abrax-488					
	Replicate			Mean	SD	SEM
	R1	R2	R3			
Neg. Ctrl	1	1	1	1	0	0
AX-A05	26.5	44.4	37.9	36.2	9.0	5.2
AX-A12	23.0	50.6	33.9	35.8	13.9	8.0
AX-A07	34.7	34.9	33.1	34.2	1.0	0.6
AX-A14	8.3	38.1	34.2	26.9	16.2	9.4
AX-A15	25.2	31.4	21.6	26.0	5.0	2.9
AX-A11	19.5	24.4	26.6	23.5	3.6	2.1
AX-A03	14.8	26.0	23.0	21.3	5.8	3.3
AX-A16	21.1	16.8	22.0	20.0	2.8	1.6
AX-A01	22.0	18.4	17.8	19.4	2.3	1.3
AX-A23	17.7	14.8	14.8	15.7	1.7	1.0
AX-A02	20.2	13.1	12.2	15.2	4.4	2.5
AX-A27	16.6	15.4	12.9	15.0	1.9	1.1
AX-A06	17.5	14.3	12.3	14.7	2.6	1.5
AX-A04	16.3	14.0	11.6	14.0	2.4	1.4
AX-A10	11.4	16.5	13.3	13.7	2.6	1.5
AX-A08	8.9	15.6	6.4	10.3	4.8	2.8

Table S2. FACS analysis of peptide binding to RiVax-488. The normalized Median Fluorescence Intensity (nMFI) for three independent replicate experiments (R1–R3), normalized to the negative control cells, is shown along with the mean, standard deviation (SD), and standard error of the mean (SEM) for this data set. This data is plotted in Figure 1.

Peptide	Rivax-488					
	Replicate			Mean	SD	SEM
	R1	R2	R3			
Neg. Ctrl	1	1	1	1	0	0
AX-A05	1.5	1.3	1.4	1.4	0.1	0.1
AX-A12	1.2	1.1	1.0	1.1	0.1	0.1
AX-A07	1.0	1.1	1.0	1.0	0.0	0.0
AX-A14	1.3	1.2	1.2	1.2	0.1	0.0
AX-A15	4.4	1.6	1.6	2.5	1.6	0.9
AX-A11	1.2	1.2	1.0	1.1	0.1	0.1
AX-A03	1.7	1.3	1.4	1.5	0.2	0.1
AX-A16	0.9	1.0	0.9	0.9	0.1	0.0
AX-A01	1.2	1.2	1.3	1.2	0.1	0.0
AX-A23	1.1	1.0	0.9	1.0	0.1	0.0
AX-A02	2.6	2.4	2.1	2.4	0.2	0.1
AX-A27	1.3	1.2	1.2	1.2	0.1	0.0
AX-A06	2.3	1.6	1.7	1.8	0.4	0.2
AX-A04	1.1	1.3	1.0	1.1	0.1	0.1
AX-A10	1.5	1.4	1.6	1.5	0.1	0.1
AX-A08	1.1	1.1	1.0	1.0	0.0	0.0

Table S3. FACS analysis of peptide binding to Streptavidin-R-Phycoerythrin (SAPE). The normalized Median Fluorescence Intensity (nMFI) for three independent replicate experiments (R1–R3), normalized to the negative control cells, is shown along with the mean, standard deviation (SD), and standard error of the mean (SEM) for this data set. This data is plotted in Figure 1.

Peptide	SAPE					
	Replicate			Mean	SD	SEM
	R1	R2	R3			
Neg. Ctrl	1	1	1	1	0	0
AX-A05	2.2	2.4	2.8	2.5	0.3	0.2
AX-A12	1.5	1.9	1.7	1.7	0.2	0.1
AX-A07	1.2	1.4	1.2	1.3	0.2	0.1
AX-A14	1.7	2.0	1.8	1.8	0.2	0.1
AX-A15	2.5	1.9	1.8	2.1	0.4	0.2
AX-A11	1.2	1.3	1.5	1.3	0.2	0.1
AX-A03	2.2	2.4	1.7	2.1	0.4	0.2
AX-A16	1.5	2.3	1.5	1.8	0.5	0.3
AX-A01	2.0	1.4	1.8	1.8	0.3	0.2
AX-A23	1.2	0.9	1.0	1.0	0.2	0.1
AX-A02	5.3	4.6	5.2	5.0	0.4	0.2
AX-A27	1.7	1.7	1.8	1.7	0.1	0.0
AX-A06	3.3	2.7	2.7	2.9	0.4	0.2
AX-A04	1.5	1.4	1.7	1.5	0.1	0.1
AX-A10	2.5	2.7	2.8	2.7	0.2	0.1
AX-A08	1.7	1.4	1.7	1.6	0.1	0.1
